# Deficiencies of Proteins C, S and Antithrombin  and Activated Protein C Resistance–Their Involvement in the Occurrence of Arterial Thromboses

**Published:** 2010-11-25

**Authors:** AM Soare, C Popa

**Affiliations:** Neurology Clinic, National Institute of Neurology and Neurovascular Diseases, BucharestRomania

**Keywords:** thrombophilia, myocardial infarction, stroke, venous thromboses

## Abstract

Deficiencies of natural anticoagulants protein C, protein S, antithrombin and activated protein C resistance are components of inherited thrombophilia. Inherited thrombophilia was defined as a genetically determined tendency towards venous thromboembolism which characteristically occurs in young age (before 40 to 45 years) without apparent causes and tend to recur. In the recent years, there has been a lot of debate about the implication of these defects in arterial thromboses (peripheral artery disease, myocardial infarction, cerebral infarction). The screening for thrombophilia is recommended for young patients with spontaneous thromboses, arterial infarctions, family history of thromboses, personal history of recurrent abortions, with thrombosis of venous dural sinuses or strokes or myocardial infarctions, in patients with venous thrombosis at unusual sites, because the diagnosis of such a disease leads to a treatment that is lifesaving [1,2].

## Introduction

Hereditary thrombophilia is defined as an enhanced inherited tendency to form intravascular thrombi, which may be arterial or venous that characteristically occurs in young age (before 45 years) and tends to recur. In 1965 the antithrombin deficiency was described and then in the early 1980s protein C and protein S deficiencies as causes of inherited thrombophilia. In 1993 the activated protein C resistance was discovered, the protease generated by the thrombomodulin–PC anticoagulant pathway to inactivate activated factors Ⅴ and Ⅷ. Activated protein C resistance (APC) is associated in 90% of cases with a point mutation in the factor Ⅴ gene (factor Ⅴ Leiden) that causes a hypercoagulable state by slowing the inactivation of activated factor Ⅴ by APC. It is the most frequent cause of inherited thrombophilia, accounting for 30% to 50% of cases [3]. These defects are acknowledged as the most important thrombophilic conditions for venous thromboembolism. Their prevalences in general population vary: 0,2%–0,4Z% for protein C deficiency, 0,2% for protein S deficiency, 0,02% for antithrombin deficiency and 4%–5%  for factor Ⅴ Leiden, 1:5.000 persons being homozygous for factor Ⅴ Leiden [3]. It is to be established if these defects are involved in arterial thromboembolism. Data regarding such an association are available mostly from case–reports and some studies. The present articol is a review of these articles.

## Clinical Manifestations

The clinical manifestations of protein C, S, antithrombin deficiencies and mutant factor V causing activated protein C resistance are similar (Table 1). 

The most common manifestations caused by protein C, S, antithrombin deficiencies and activated protein C resistance are deep–vein thrombosis of the lower limbs, that accounts for approximatively 90% of all venous thrombotic episodes and pulmonary embolism, though venous thromboses can occur in other venous sites (upper limbs veins, hepatic vein, cerebral veins, retinal vein, mesenteric vein), but these are rare (5% of venous thrombosis in patients with these defects).

Chronic sequelae can be extremely debilitating, because of the postthrombotic syndrome, that can affect up to 20% of the patients [5]. 

The relative risks of a first episode of venous thrombosis due to these types of inherited thrombophilic defects are presented in the following table, as published in the retrospective study LEIDEN TROMBOPHILIA STUDY (LETS) and in the prospective study LITE, that included over 20.000 subjects [6,7].

**Table 1:** Clinical features of patients with inherited deficiencies of antithrombin, protein C, protein S and activated protein C resistance [4] (*All these features are less evident in patients with activated protein C resistance, who appear to be less severely affected clinically)

VENOUS THROMBOEMBOLISM (>90% OF CASES) DEEP VEIN THROMBOSIS OF LOWER LIMBS (common) PULMONARY EMBOLISM (common) SUPERFICIAL THROMBOPHLEBITIS MESENTERIC VEIN THROMBOSIS (rare, but characteristic)
CEREBRAL VEIN THROMBOSIS (rare, but characteristic)
FAMILY HISTORY OF THROMBOSIS *
FIRST THROMBOSIS USUALLY IN YOUNG AGE (< 40 YEARS) *
FREQUENT RECURRENCES *
NEONATAL PURPURA FULMINANS (homozygous protein C and protein S deficiency

**Table 2:** Thrombophilia: relative risks of a first venous thrombosis, as published by the retrospective Leiden Thrombophilia Study (LETS) and the prospective cohort study LITE [8] (*An accurate determination of risk is difficult because of low prevalence)

Risk factor	Retrospective VTE risk (LETS study)	Prospective VTE risk (LITE study)
Factor V Leiden (heterozygote)	8.1	3.7
Factor V Leiden (homozygote)	80	24
Prothrombin 20210A	2.8	1.9
Protein C deficiency*	3.1	3.4
Protein S deficiency*	0.8	not available
Antithrombin deficiency*	5.0	not available

After a first episode of deep vein thrombosis, reccurents can occur in patients with or whithout known thrombophilic conditions, the highest risk being in the first 6 months. A cumulative incidence of deep vein thrombosis up to 25% after 5 years of clinical follow–up is frequentlly quoted [9], though Leiden Thrombophilia Study reported a cumulative incidence of 12,4% after 5 years [8].

More rare sites for venous thromboses, but characteristic, are: cerebral vein thrombosis, splanchnic veins thrombosis–extrahepatic portal vein thrombosis, Budd–Chiari syndrome and mesenteric vein thrombosis, upper–extremity deep vein thrombosis  [10]. 

Consequently, thrombophilic defects are responsible for obstetrical complications: early pregnancy loss, late pregnancy loss, pre–eclampsia,  intrauterine growth restriction, and abruptio placentae  [11,12,13].

Because numerous cases of patients with personal history of previously venous thrombosis that had arterial thrombosis have been reported, in the recent years there have been a lot of studies to establish whether pacients with thrombophilia and antecedents of venous thromboembolisms have a higher risk of arterial thrombosis [14,15]. Such a study included 1081 consecutive patients (649 F/432 M, age range 16–93) registered with venous thromboembolism in MAISTHRO database (MAin–ISar–THROmbosis). Thrombophilia screening tested the presence of factor Ⅴ Leiden, G20210A prothrombin gene mutation, antiphospholipid antibodies, factor Ⅶ, protein C, protein S and antithrombin activities. From all of the patients, 40 (3.7%) had a prior myocardial infarction (MI) and 41 (3.8%) had a  stroke. Only the presence of lupus anticoagulant was found statistically significant.The conclusion was that the cumulative incidence of arterial thrombotic events is low for those with antecedents of venous thrombosis and inherited thrombophilia doesn't seem to substantially increase the risk of arterial thromboses [16]. Another study that included members of protein C, S and antithrombin defficient families also proved that subjects with previous venous thrombosis had a similar risk for arterial thrombosis as those without antecedents of venous thrombosis, so there seems to be no association between venous thrombosis and the subsequent arterial thrombosis [17].

Only some studies and case reports have discussed the implication of trombophilic defects in arterial thrombosis. The results are controversial. In one cohort family study, the arterial events were diagnosed in 8% of the 144 subjects with protein C or S deficiencies and 1% from the 94 subjects with antithrombin deficiency [18]. In another ample study on carriers of familial thrombophilia (the European Prospective Cohort on Thrombophilia [EPCOT] study), overall annual incidences of myocardial infarction and/or ischemic stroke after 20 years of age were 0.15%, 0.18Z%, and 0.15% in subjects with protein S (n=111), protein C, (n=150), and antithrombin deficiency (n=92), respectively [19]. A more recent study reported annual incidences of myocardial infarction and/or ischemic stroke of 0.32% (protein S), 0.32% (protein C), and 0.21% (antithrombin) in deficient subjects and 0.19% in non–deficient subjects >20 years of age [16]. In a case–control study, arterial thrombosis was recorded more frequently in 88 cases with protein S, protein C, or antithrombin deficiency (19% arterial thrombosis) compared with control subjects with venous thromboembolism without these deficiencies (1% arterial thrombosis) [20].

Another case–control study reported significantly lower plasma levels of activated protein C in young patients with myocardial infarction (n=231) compared to healthy controls (n=231)[21]. In a Japanese study, subjects with established inherited protein C deficiency and either myocardial infarction (n=10) or ischemic stroke (n=11) were on average 11 and 7 years younger at the onset of the myocardial infarction or ischemic stroke, respectively, than control subjects with myocardial infarction (n=42) or ischemic stroke (n=48) with normal protein C levels [22]. The prospective epidemiological Atherosclerosis Risk in Communities (ARIC) study reported that plasma protein C appeared to have a protective role against ischemic stroke but not against myocardial infarction [23]. 

On the other hand, in other case–control studies, prevalences of these deficiencies were similar between cases with ischemic stroke [18,24] or myocardial infarction [18,25,26] and matched controls, even for young patients [25,26]. Moreover, in the ARIC study, the low levels of plasma protein C and antithrombin were not related to coronary heart disease [23]. The lack of association between protein S, protein C, or antithrombin deficiency and risk for arterial thromboses in these studies could be explained by the low prevalence of these hereditary deficiencies in the general population. More common, but weaker, thrombophilic defects (i.e., factor Ⅴ Leiden, the prothrombin G20210A mutation) were more frequently related to myocardial infarction [25,26,27]  and/or overall coronary disease (i.e., myocardial infarction or coronary stenosis) [27]. In addition, it could be speculated that protein S, protein C, or antithrombin deficiency in these studies might have been acquired rather than hereditary, considering that acquired deficiencies are more prevalent. The latter have a high prevalence (up to 4%) in control subjects [24].

A retrospective family cohort study published in 2008 performed a retrospective follow–up study to assess the risk of arterial thrombosis (myocardial infarction, ischemic stroke, transient ischemic attack, or peripheral artery disease) in a large series of 468 (52% women, average age, 46 ± 17 years) protein S–, protein C–, or antithrombin–deficient subjects, relatives of patients with venous thrombosis compared with nondeficient family members [17]. Of the 468 subjects, 35% had protein S deficiency, 39% protein C deficiency and 26% had  antithrombin deficiency. These subjects were clinically followed up for a 12 year period and 11% of the PS deficient patients, 11% of the PC deficient patients and 8% of the antithrombin deficient patients had arterial thrombosis. Utterly, the lifetime risk of arterial thrombosis was   2–fold higher in subjects with any deficiency (i.e., protein S, protein C, or antithrombin) compared to non#x2013;deficient subjects. The high risk of arterial thrombosis conferred by any deficiency was evident only until   55 years of age, a 5–fold risk increase. For separate deficiencies, the risks were 4.6– (95% CI, 1.1 to 18.3), 6.9– (95% CI, 2.1 to 22.2), and 1.1– (95% CI, 0.1 to 10.9) fold higher in protein S–, protein C–, and antithrombin–deficient subjects, respectively, before 55 years of age. Subjects with any of the 3 deficiencies were on average by 11 years younger at the onset of ATE compared with nondeficient subjects. Interestingly, only protein S and protein C deficiencies were related to ATE before 55 years of age. Antithrombin deficiency was not related to a significantly increased risk either before 55 years of age or thereafter. A high risk for arterial thrombosis conferred by protein S or protein C but not antithrombin deficiency was also reported earlier [28,29]. It could be speculated that the higher risk for arterial thrombosis in subjects with protein C deficiency could be ascribed to the potent cytoprotective effects of the protein C pathway [30].Protein S deficiency, rather than antithrombin deficiency, is associated with artherial thrombosis because of the synthesis of protein S by endothelial cells, whereas antithrombin is synthesized by hepatocytes. Endothelial injury as a trigger of thrombosis may be enhanced by a preexisting defect in protein S synthesis at the site of the injury. Some cytoprotective effects have been attributed to protein S [31]. 

## Conclusion

Thrombophilia is a serious inherited disease, that expose the carrier to life-threatening arterial and venous thrombosis.The incidence of thromboses for the defficient persons varies, some of them could never display a trombosis, others can have recurrent thromboses. This depends on the  genotype in case, the coexistence of other genetic defects and the influence of environmental risk factors, such as trauma, surgical procedures, oral contraceptive use and pregnancy. Faced with a case of thrombosis without known risk factors, the screening of thrombophilia must be performed in order to procede to the adequate anticoagulant treatment.
